# Cooper pair splitting in parallel quantum dot Josephson junctions

**DOI:** 10.1038/ncomms8446

**Published:** 2015-07-01

**Authors:** R. S. Deacon, A. Oiwa, J. Sailer, S. Baba, Y. Kanai, K. Shibata, K. Hirakawa, S. Tarucha

**Affiliations:** 1 Advanced Device Laboratory, RIKEN, Wako 351-0198, Japan; 2 Center for Emergent Matter Science (CEMS), RIKEN, Wako 351-0198, Japan; 3 The Institute of Scientific and Industrial Research, Osaka University 8-1 Mihogaoka, Ibaraki 567-0047, Japan; 4 Department of Applied Physics, The University of Tokyo, 7-3-1 Hongo, Bunkyo-ku, Tokyo 113-8656, Japan; 5 Institute of Industrial Science, The University of Tokyo, 4-6-1 Komaba, Meguro-ku, Tokyo 153-8505, Japan; 6 INQIE, The University of Tokyo, 4-6-1 Komaba, Meguro-ku, Tokyo 153-8505, Japan; 7 JST CREST, 4-1-8 Hon-cho, Kawaguchi-shi, Saitama 332-0012, Japan; 8 QPEC, The University of Tokyo, 7-3-1 Hongo, Bunkyo-ku, Tokyo 113-8656, Japan

## Abstract

Devices to generate on-demand non-local spin entangled electron pairs have potential application as solid-state analogues of the entangled photon sources used in quantum optics. Recently, Andreev entanglers that use two quantum dots as filters to adiabatically split and separate the quasi-particles of Cooper pairs have shown efficient splitting through measurements of the transport charge but the spin entanglement has not been directly confirmed. Here we report measurements on parallel quantum dot Josephson junction devices allowing a Josephson current to flow due to the adiabatic splitting and recombination of the Cooper pair between the dots. The evidence for this non-local transport is confirmed through study of the non-dissipative supercurrent while tuning independently the dots with local electrical gates. As the Josephson current arises only from processes that maintain the coherence, we can confirm that a current flows from the spatially separated entangled pair.

## Abstract (web summary)

Spin-entangled electron pairs are one possible resource for future solid-state quantum information processing systems. Here, the authors directly prove spin entanglement between two electrons that had previously been a Cooper pair in a superconducting lead but were split using two quantum dots.

## Graphical abstract

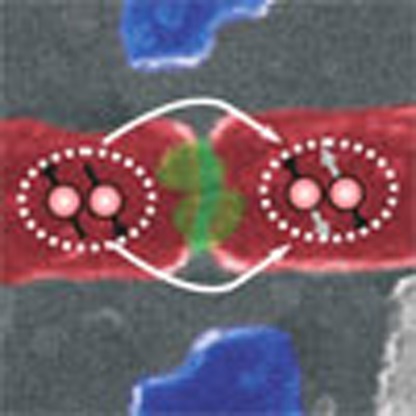

Since the famous Gedankenexperiment of Einstein, Podolsky and Rosen^1^ and later experimental demonstration of quantum non-locality^2^, the phenomenon of quantum entanglement has been accepted as a fundamental feature of quantum mechanics. Quantum entanglement has been identified as a useful property for application in both computation and communication^3^. While entanglement itself is ubiquitous, the preparation and isolation of useful entangled states such as a maximally entangled Einstein–Podolsky–Rosen (EPR) pair is non-trivial. Most headway in this area has been made in quantum optics in which the sources of EPR pairs are well established and already applied for long distance quantum communication^4^. In the solid state, an EPR source for entangled electrons is highly desirable as a circuit element for a quantum information processor allowing the production of EPR pairs to be used for the teleportation of qubit states across a chip. One attractive proposal for a solid-state EPR source utilizes a superconductor as a natural reservoir for EPR pairs, which could be extracted and separated on-demand^5^,^6^,^7^,^8^,^9^,^10^,^11^. In a BCS superconductor, a Cooper pair is composed of two electrons with opposite momenta and spin singlet state paired through an attractive electron–electron interaction mediated by phonons. Intuitively, the spatial extent of the Cooper pair given by the BCS coherence length *ξ*_0_ imposes a restriction on the possible separation between contacts into which the Cooper pair could be split. In practice, however, the probability of splitting the Cooper pair into two spatially separated leads is a function of both *ξ*_0_ and the Fermi wavelength *λ*_F_ (refs 6, 12, 13). Most experimental studies to date have used nanotubes^14^,^15^ or nanowires^16^,^17^ in which the proximity effect in the sections of nanotube or wire between the quantum dots (QDs) can lead to an increased probability for splitting^18^. Despite the limitation on the efficiency of splitting based on the probability of separating the Cooper pair, several groups have reported indications of highly efficient splitting of Cooper pairs in QD Y-junction devices^14^,^15^,^16^,^17^ with recently reported efficiency approaching unity^15^. The strong electron–electron interaction on the QDs suppresses the tunnelling of Cooper pairs through a single dot and therefore reinforces the process of crossed Andreev reflection allowing the separation of Cooper pairs into the two metal leads. Measurements have probed the Cooper pair splitting through observation of non-local charge signals^14^,^15^,^16^ and correlation of the current fluctuations^17^. However, in these first measurements, the entangled spin state is not directly confirmed.

In this work, we study a device in which two QDs are placed in close proximity within a nanogap between two superconducting leads, a system so far only considered in a small number of theoretical studies^19^,^20^,^21^. By measuring the supercurrent in this device, we detect its enhancement when Cooper pairs from one lead are split between the two QDs and then recombined in the second lead.

## Results

### Device details

**Figure 1:**
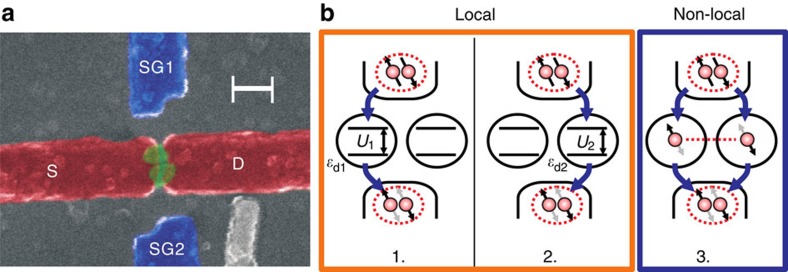
Sample image and schematic. (**a**) False colour scanning electron microscope image of the device with side-gates (SG) and source (S) and drain (D) electrodes indicated. Scale bar, 150 nm. (**b**) Schematic of the three lowest order (fourth order) Cooper pair tunnelling processes. In processes (1) and (2), the Cooper pair tunnels through a single QD. In process (3), the Cooper pair is split through both QDs and a non-local entangled state (EPR state) is achieved. In each process, the specifics of the tunnelling and the parity of the QD occupation may result in reversal of the spin order of the Cooper pair giving a negative contribution to the Josephson energy of the junction.

The device studied consists of two self-assembled InAs QDs contacted with Ti/Al (3/150 nm) source and drain electrodes, Fig. 1. In our device, each QD can be independently tuned using voltages *V*_sg1_ and *V*_sg2_ applied to local side-gates allowing control of the local energy levels *ɛ*_d1_ and *ɛ*_d2_ for QD1 and QD2, respectively. A voltage *V*_bg_ applied to a global backgate allows tuning of the occupation of both QDs simultaneously and allows us to probe a wider range of charge states in both QDs. If the spatial separation of the contact to the two QDs is less than the superconducting coherence length (*ξ*_0_), we may expect crossed Andreev reflection processes where a single Cooper pair is separated into the two QDs, as depicted in Fig. 1b. The non-dissipative Josephson current that flows in the presented system is captured in the Josephson energy of the junction (*E*_J_), which indicates the potential energy stored and is proportional to the critical current (*E*_J_∝*I*_c_). *E*_J_ can be written as a sum of three components.


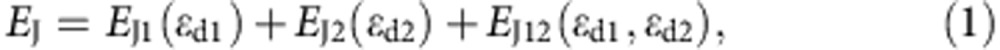


where *E*_J1_(*ɛ*_d1_) and *E*_J2_(*ɛ*_d2_) are the contributions to *E*_J_ for the local tunnelling of a Cooper pair through QD1 and QD2, respectively, and *E*_J12_(*ɛ*_d1_, *ɛ*_d2_) is the contribution for non-local transport processes in which a Cooper pair is split between the two QDs and then recombined as shown in Fig. 1b. Note that *E*_J12_ is a function of the energy levels of both QDs. The local processes are suppressed by the on-site charging energy of each QD (*U*_1_ and *U*_2_), which prevents the tunnelling of two electrons. The non-local process is unaffected by the on-site charging energy but its probability decreases with the separation of the contacts to the two QDs (the distance over which the Cooper pair must be separated inside the contact).

The proposed scheme to detect the contribution to the Josephson current arising from the non-local processes is to tune one QD to be OFF resonance such that only the other QD contributes to the transport and use measurements under these conditions as a background or reference for comparison with transport when both QDs are ON or near resonance. One complication of this treatment is that the sign of each component of the Josephson energy maybe positive or negative depending on the specifics of the QD occupation and the number and parity of the orbital states involved in the transport^22^. The measurement of the switching current of the device only provides an absolute measurement of the Josephson current. By measuring the supercurrent, we detect only processes that result in the phase coherent tunnelling of a pair of electrons in the singlet state. In practice, it is possible for the two QDs to be occupied by electrons in the triplet state, which cannot contribute to the supercurrent and will therefore not be measured.

### Normal state characterization

**Figure 2:**
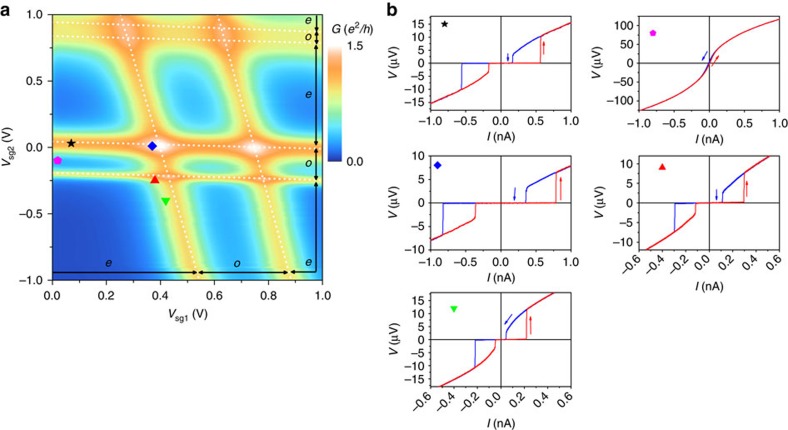
Device characterization. (**a**) Plot of the differential conductance (d*I*/d*V*_sd_) as a function of *V*_sg1_ and *V*_sg2_ with *V*_sd_=0 V, *B*=160 mT applied out-of-plane and *V*_bg_=0 V. (**b**) Example *V*(*I*) traces measured at the the points indicated in **a**. Arrows indicate the current sweep direction.

Before examining the superconducting transport, we discuss transport in the normal state when a magnetic field (*B*) is applied perpendicular to the sample surface. In this configuration, the critical magnetic field of the aluminium leads is *B*_c_∼110 mT. An example of the normal state charging stability diagram is presented in Fig. 2a for *B*=160 mT. Despite the physical proximity of the two QDs, we observe negligible interdot tunnel capacitive coupling, which maybe expected to vary with the specific charge states but is evaluated as being <30 μeV in the regions that we have studied and is therefore smaller than the superconducting energy gap (see Supplementary Fig. 1 and Supplementary Note 1). We conclude therefore that the nanogap electrodes very effectively screen the two QDs. The side-gate electrodes are able to independently tune the occupation of the two QDs with only a small effect on QD1 from *V*_sg2_ and vice versa. Both QDs are operated in the many electron regime (a few tens of electrons) for which the energy level spacing and charging energy vary with orbital state^23^. Using stability plots measured under various conditions, we extract typical ranges for the charging energies of *U*_1_∼2−4 meV and *U*_2_∼2−5 meV. Similarly, typical energy level spacings are evaluated in the ranges *δɛ*_d1_>3–4 meV and *δɛ*_d2_∼1–4 meV. By tuning the charge state using the backgate, we are able to realize a wide range of different QD-lead tunnel coupling (Γ), evaluated in the normal state from the width of Coulomb peaks^24^, in the range Γ=0.1–1.5 meV. Typically, we find that *δɛ*_d1_,*U*
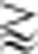
Γ>Δ such that the device is in the intermediate coupling regime^25^. The large energy level spacing and charging energy compared with the Aluminium superconducting energy gap (Δ∼130 μeV, see Supplementary Fig. 2 and Supplementary Note 2) indicates that a single-level picture is a reasonable approximation for the system. No signature of interdot tunnel coupling is seen for any of the charge states we have studied and so a lower bound for the possible interdot coupling can be estimated from the lowest dot-lead tunnel couplings measured indicating *γ*_interdot_<<0.1 meV and consequently *γ*_interdot_<<Δ. The charging energies and other parameters may alter slightly when the side-gates are swept^23^,^24^,^26^; however, these changes are typically smooth and small for this sample over the gate ranges we are considering. At high magnetic fields, we observe clear Aharanov–Bohm oscillations, which are consistent with the small dimensions of the junction (Supplementary Fig. 3 and Supplementary Note 3) and indicate that coherent transport through the parallel QDs is possible despite the short coherence length in the metal contacts. We also observe an anomalous conductance feature when both QDs are tuned ON resonance in the normal state, which is discussed in Supplementary Figs 4–8 and Supplementary Note 4.

### Superconducting transport

**Figure 3:**
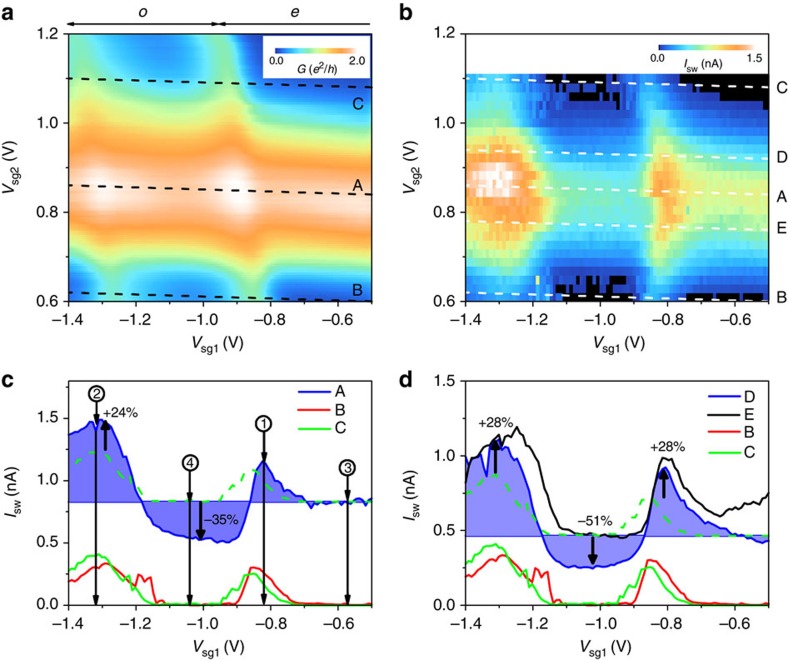
Superconducting state transport measurement A. (**a**) d*I*/d*V*_sd_ plotted as a function of *V*_sg1_ and *V*_sg2_ with *V*_bg_=0 V and *B*=160 mT applied out-of-plane. Labels *o* and *e* indicate even and odd electron occupation respectively. (**b**) *I*_sw_ plotted as a function of *V*_sg1_ and *V*_sg2_ with *V*_bg_=0 V and *B*=0*T*. (**c**) *I*_sw_ extracted along lines A, B and C in plot (**b**). The background indicated for trace A is taken as the value of *I*_sw_ measured at point 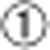 (*V*_sg1_=−0.57 V) where QD1 is OFF resonance. Points 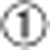 (*V*_sg1_=−1.32 V), 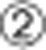 (*V*_sg1_=−0.824 V) and 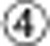 (*V*_sg1_=−1.04 V) are discussed in the main text. The dashed line indicates trace C offset by the current at point 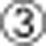 of trace A. Arrows and percentages indicate the approximate increase (decrease) in *I*_sw_ evaluated by the deviation of trace A from the dashed reference line. (**d**) *I*_sw_ extracted along lines B, C, D and E in plot (**b**). The dashed line indicates trace C offset by a background *I*_sw_ taken at a point where QD1 is OFF resonance in trace D. Arrows and percentages indicate the relative enhancement and suppression of *I*_sw_ evaluated by the deviation of trace D from the dashed reference line. Lines D and E are taken to be near resonance with QD2 and show a larger relative enhancement compared with the both QD ON resonance condition (line A in **c**).

**Figure 4:**
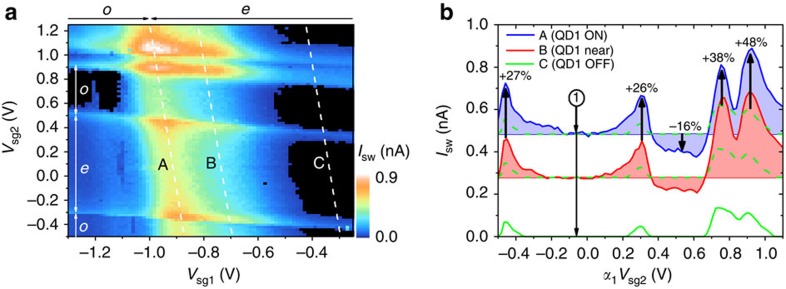
Superconducting state transport measurement B. (**a**) *I*_sw_ as a function of *V*_sg1_ and *V*_sg2_ at *V*_bg_=0 V and *B*=0 T. Dashed lines indicate the traces A, B and C plotted in **b**. Labels *e* and *o* indicate even and odd electron occupation, respectively. (**b**) *I*_sw_ traces as a function of *α*_2_(*V*_sg1_)*V*_sg2_ extracted along lines indicated in plot **a**. The variable *α*_2_ corrects for the influence of *V*_sg1_ on QD2 for easier comparison of the traces. The backgrounds for the colour fill of traces A and B are taken as *I*_sw_ measured at point 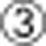 (*α*_2_*V*_sg2_=−0.059) where *I*_sw_ with QD2 OFF resonance is zero (seen in trace C). The dashed lines indicate trace C offset (again by the *I*_sw_ and point 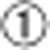) for comparison with trace A and trace B. Arrows and percentages indicate the approximate increase (decrease) in *I*_sw_ when QD1 and QD2 are ON or near resonance (QD1 ON/near resonance and QD2 with odd occupation).

**Figure 5:**
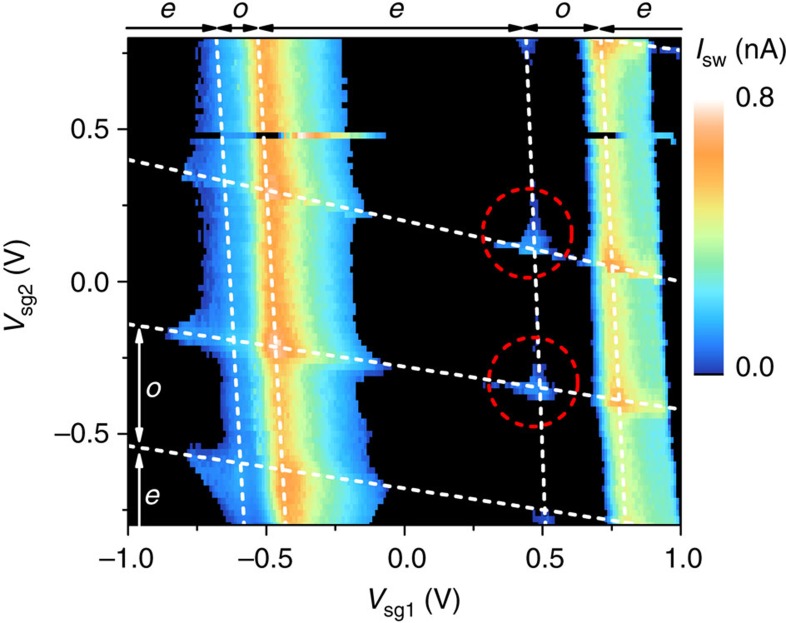
Superconducting state transport measurement C. *I*_sw_ as a function of *V*_sg1_ and *V*_sg2_ with *V*_bg_=1.6 V and *B*=0 T. Dashed lines indicate the positions of Coulomb charging peaks. Labels *o* and *e* indicate regions of odd and electron occupation where known. Circled regions indicate the points discussed in the text at which QD1 and QD2 are both ON resonance and the majority of supercurrent arises from the non-local tunnelling.

We now consider the measurement of the junction critical current and anomalous transport features, which we attribute to the non-local Cooper pair tunnelling in the double QD Josephson junction. Typical *V*(*I*) traces at *B*=0*T* are shown in Fig. 2b. For the ranges of side-gate bias and backgate bias studied in this report, the junction is predominately underdamped indicating that the dissipation in the circuit is weak compared with the phase changes (fluctuations) across the junction. *V*(*I*) traces display characteristic hysteretic switching between Josephson current and normal dissipative current branches. The transport is characterized by a switching current (*I*_sw_), which is taken as proportional to the junction critical current and a retrapping current (*I*_r_). Recent studies indicate that the hysteresis likely arises from the heating of the junction in the normal state rather than effects related to the dynamics of the junction^27^. The signature of the non-local processes has been studied in several different transport regions with different relative couplings of the individual QDs (see also Supplementary Fig. 9 and Supplementary Note 5). Where indicated, the even and odd electron occupation of each QD has been confirmed either by observation of the Zeeman splitting of Coulomb peaks or features in the superconducting transport associated with the odd electron occupation such as asymmetric supercurrent when the gate is swept across a Coulomb peak^24^ (see Supplementary Figs 10 and 11 and Supplementary Note 6). In the remaining figures (Figs 3, 4, 5), we study the measured *I*_sw_ in the superconducting state as a function of *V*_sg1_ and *V*_sg2_. The measured *I*_sw_ is extracted at each data point from *V*(*I*) plots as shown in Fig. 2b. The signatures of the non-local or split Copper pair tunnelling are discussed in the section which follows.

## Discussion

Consider the measurements presented in Fig. 3 collected for a region of the gate parameter space in which QD2 is relatively strongly coupled compared with QD1. To allow study of the non-local current, we extract traces along lines A–E, which are parallel with the Coulomb charging peaks for QD2, and use these to compare *I*_sw_ with both QDs ON resonance (trace A) to *I*_sw_ with only one QD ON resonance (traces B and C). Initially, we consider the Josephson current when both QDs are tuned to be far OFF resonance. When one QD is tuned to be OFF resonance, the Josephson current arising from local Cooper pair tunnelling through that QD is negligible as a result of the large *U*. The non-local processes are most efficient when both QDs are near resonance so by tuning both QD far from resonance the non-local contribution can be minimized or even zero. Indeed, we find in our device that with both QDs in the Coulomb blockade we can detect no supercurrent. Next we consider traces B and C in Fig. 3c, which are taken for the condition that QD2 is far OFF resonance. Here when QD1 is also away from resonance (in the Coulomb blockade), for example, point 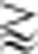, we observe that *I*_sw_=0 indicating that *E*_J1_, *E*_J2_, *E*_J12_∼0 as previously discussed. Similarly at point 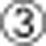, where QD1 has an odd electron occupation but is OFF resonance, we observe that *I*_sw_=0. When *V*_sg1_ is tuned QD1 is brought into or ON resonance at points 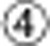 and 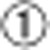. At these points, a peak in *I*_sw_ is observed as the local tunnelling processes allow a supercurrent to flow. As QD2 is OFF resonance *E*_J2_∼0, and so the supercurrent is given by *E*_J_=*E*_J1_+*E*_J12_. We may naively assume that *E*_J12_ is negligible such that the measured *I*_sw_ largely reflects the local transport through QD1.

We now consider the case where QD2 is ON resonance as shown in trace A. Here tuning *V*_sg1_ shifts QD1 ON and OFF resonance allowing us to observe the effects on the supercurrent. When QD1 is brought ON resonance peaks in *I*_sw_ are observed that arise from all available transport processes both local and non-local. Furthermore, a reduction in *I*_sw_ is seen when QD1 has an odd electron occupation in the Coulomb blockade, a condition for which in traces B and C no supercurrent was detected. We take the level of *I*_sw_ at point 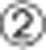 of trace A (selected as QD1 is OFF resonance with even occupation) as a reference, indicated by the colour fill in Fig. 3c, to better visualize the anomalous features. We stress, however, that at point 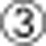, we cannot rule out a finite non-local contribution *E*_J12_ and so in our analysis we are unable to evaluate absolute contributions from the non-local processes (or confidently select a background level for the local transport through QD2). We assume that the influence of local transport through QD1, *E*_J1_(*ɛ*_d1_), is captured in traces B and C where QD2 is OFF resonance. As we observe in traces B and C that *I*_sw_=0, and therefore *E*_J1_∼0, in the QD1 Coulomb blockade we can attribute features in this region when QD2 is ON resonance as arising only from *E*_J_=*E*_J2_+*E*_J12_. As there is a clear variation of *I*_sw_ when the occupation parity of QD1 is altered, we can write that at point 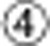 of trace A the Josephson energy is given by 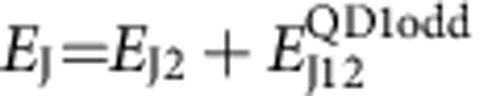 and at point 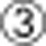 it is given by 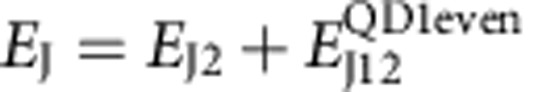. At point 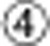 of trace A, we observe a ∼35% reduction of *I*_sw_ compared with reference point 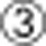. We are able to subtract out the local transport processes through QD2 by finding the difference between *I*_sw_ at points 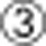 and 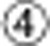 resulting in 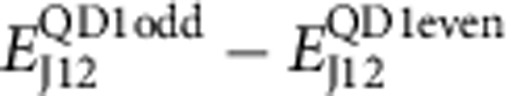, which gives an order estimate of a contribution of the non-local process to supercurrent of Δ*I*_sw_∼0.3 nA. As previously mentioned, it is possible that the non-local process results in a negative contribution to the Josephson energy either due to occupation parity or orbital parity. In Supplementary Figs 12–16 and Supplementary Note 7, we consider the possibility that the tuning of one QD ON/OFF resonance alters the dissipation in the circuit causing a change in the measured *I*_sw_. By measuring the hysteresis in the *V*(*I*) traces we investigate changes due to the variation of the local electromagnetic environment. We observe that while the hysteresis is altered by tuning the device gates the features are not correlated with the enhanced and suppressed *I*_sw_ identified as a signature of the non-local transport. We still however cannot exclude a small effect due to changes in the dissipative environment of the junction due to one QD acting as a tunable shunt for the second QD.

We can also consider *I*_sw_ at points 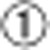 and 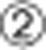 where both QDs are ON resonance. Through comparison with traces B and C with trace A, we observe a ∼24% increase in *I*_sw_ at point 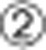. At point 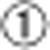, however, *I*_sw_ is approximately equal to the sum of OFF resonance *I*_sw_ for each QD. While we cannot extract the non-local contribution in the reference data at these points, we do not expect the local contributions to alter indicating that the enhancement of *I*_sw_ originates from a large increase in *E*_J12_ with both QDs ON resonance at point 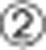. We also consider the case of QD2 near resonance in Fig. 3d and observe that the relative contribution from non-local processes is increased as the local transport through QD2 is reduced when tuned away from resonance.

To show that the features are reproducible, we consider another region of the gate parameter space, presented in Fig. 4a, which is measured on a different cooldown of the same device. In this case, a wider gate range is studied in which QD1 exhibits two Coulomb peaks with a relatively stronger coupling than QD2, giving higher local supercurrents than QD2. As in our previous analysis, we extract traces A–C taken with QD1 ON (trace A and B) and OFF (trace C) resonance, Fig. 4b. In trace C with QD1 OFF resonance, we again only observe finite supercurrent under conditions for which QD2 is brought ON or near resonance. In traces A and B, we again plot data with a colour fill to a background selected as the even occupation Coulomb blockade of QD2 taken at point 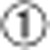. We observe that for some regions with odd electron occupation *I*_sw_ is reduced indicating the influence of the non-local transport processes as the local transport through QD1 is not influenced by the parity of QD2. We also observe the enhancement of *I*_sw_ when both QDs are ON or near resonance but more clearly than in Fig. 3c. The enhanced and reduced supercurrent again provide clear signature of the non-local Cooper pair tunnelling process. The parity effect of the OFF resonance QD on the non-local Cooper pair tunnelling as observed in Figs 3 and 4 appear pronounced when the OFF resonance QD is weakly coupled compared with the other ON resonance QD. For both QDs, approximately similarly coupled to the leads we also observe the parity effect albeit less pronounced. Finally, we consider the region in Fig. 5 that shows *I*_sw_ evaluated for a wide range of side-gate voltages showing a variation in switching current for different Coulomb peaks. As previously discussed, the coincidence of the resonance conditions with both QDs results in an enhanced supercurrent for the junction. The effect here is particularly prominent for the two circled coincident resonance conditions as for the relevant QD1 and QD2 Coulomb peaks for these crossings we observe no supercurrent away from the crossing point. This indicates that the local transport of these states is completely suppressed. The peak observed in the supercurrent when both QDs are ON resonance can then be considered to arise from only non-local processes in this case.

In summary, we fabricate a single Josephson junction containing two InAs self-assembled QDs in parallel and close proximity such that a Josephson current may flow by splitting of single Cooper pairs between the two QDs. This non-local transport path that indicates the spatially split entangled pair is identified from the comparison of the Josephson current measured when the QDs are tuned ON/OFF resonance using local gates. Experimentally, we have observed similar signatures in various regions and devices in a wide range of asymmetry in the tunnel couplings of QD1 and QD2. Further studies of the non-local spin entangled state maybe achieved by including an additional pilot Josephson junction or using superconducting material with high critical magnetic fields to allow manipulation of the superconducting phase difference across the double dot Josephson junction^19^,^21^.

## Methods

### Fabrication details

The InAs self-assembled QDs were grown by molecular beam epitaxy on a (100)-oriented GaAs substrate. The growth layers on the substrate consist of a 200-nm thick degenerately Si-doped layer, used as the backgate, followed by a 100-nm thick *A*l_0.3_*G*a_0.7_As barrier layer and a 200-nm thick undoped GaAs buffer layer. The InAs QDs were formed through the Stranski–Krastanov growth mode from a wetting layer of ∼4 monolayers of InAs which results in a mixed phase of large and small QDs. To accurately contact two QDs in close proximity, we first deposit arrays of Ti/Au alignment markers using electon beam lithography and evaporation. We then use atomic force microscopy to identify suitable pairs of QDs. Finally the targetted pairs were contacted and side-gates were added using a single electron beam lithography step. To achieve transparent superconducting contacts, the QDs are exposed to a weak Argon plasma *in situ* within the processing chamber before deposition of (3/150 nm) Ti/Al contacts by electon beam evaporation.

### Measurement details

Measurements are performed in a dilution refridgerator with a 30-mK base temperature and equipped with a superconducting magnet. The measurement lines were filtered with a series of room temperature and cold filters. These include commercial pi-filters (minicircuits BLP-1.9+) at room temperature, and custom-made copper powder and RC filters mounted at the mixing chamber stage of the fridge. Following the filters, the sample itself was mounted in an rf-tight copper enclosure.

## Additional information

**How to cite this article**: Deacon, R. S. *et al.* Cooper pair splitting in parallel quantum dot Josephson junctions. *Nat. Commun.* 6:7446 doi: 10.1038/ncomms8446 (2015).

## Supplementary Material

Supplementary Information Supplementary Figures 1-16, Supplementary Notes 1-7 and Supplementary References
