# Erratum: Cooper pair splitting in parallel quantum dot Josephson junctions

**DOI:** 10.1038/ncomms10299

**Published:** 2015-12-14

**Authors:** R. S. Deacon, A. Oiwa, J. Sailer, S. Baba, Y. Kanai, K. Shibata, K. Hirakawa, S. Tarucha

Nature Communications 6: Article Number: 744610.1038/ncomms8446 (2015); Published 07012015; Updated 12142015

The financial support for this Article was not fully acknowledged. The Acknowledgements should have included the following:

ST acknowledges financial support from Grant-in-Aid Research S No. 26220710.

