# Supplementary material for: Cooper pair splitting in parallel quantum dot Josephson junctions
Source: Nat Commun. 2015 Jul 1;6:7446. doi: 10.1038/ncomms8446 (PMC4506998; doi:10.1038/ncomms8446)
Supplement: Supplementary Information — Supplementary Figures 1-16, Supplementary Notes 1-7 and Supplementary References [file ncomms8446-s1.pdf]

Supplementary Figures

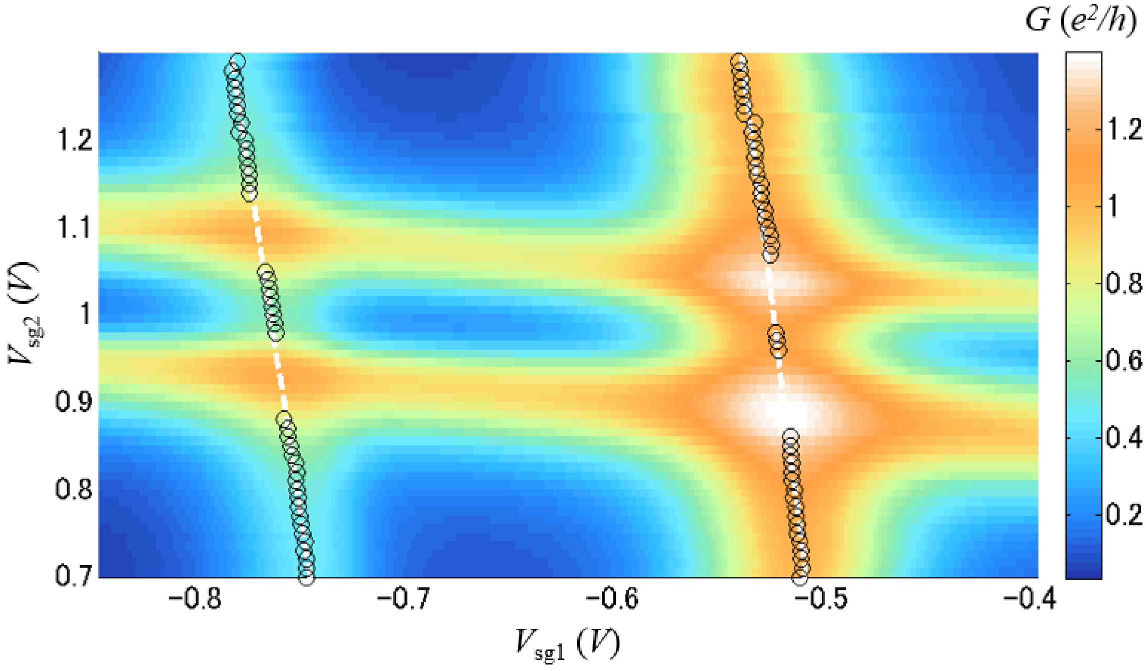

Supplemental Figure 1: Estimation of inter-dot capacitance. Differential conductance as a function of V_{sg1} and V_{sg2} for $V_{\text{bg}} = 1.6\,V$, $V_{\text{sd}} = 0\,V$ and leads in the superconducting state ($B = 0\,T$). White circles mark Coulomb peak positions for QD1 extracted using lorentzian fitting and dashed lines are linear fits through all Coulomb peaks. In this gate region as with others studied no obvious offset of the Coulomb peak position is observed for QD1 when QD2 is charged and vice versa.

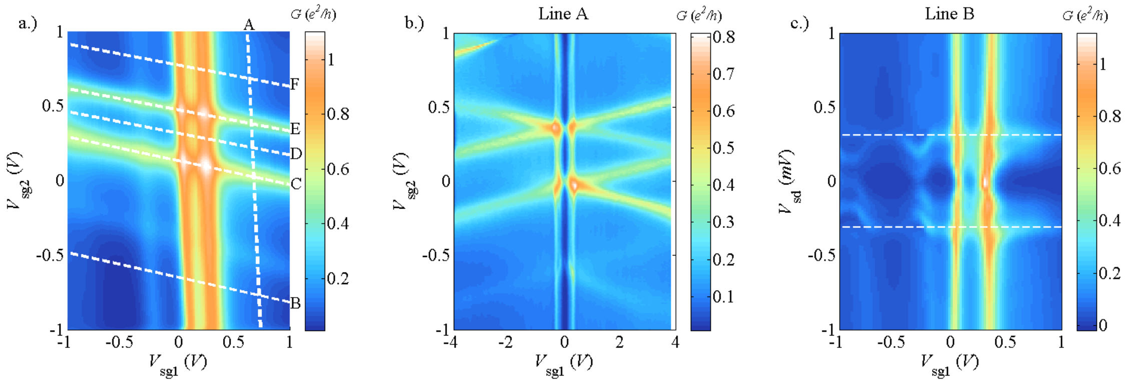

Supplemental Figure 2: Dissipative transport in the superconducting state. (a) Plot of the differential conductance (dI/dV_{sd}) as a function of V_{sg1} and V_{sg2} with $V_{\mathrm{sd}} = 0\,V$, $B = 160\,mT$ applied out-of-plane and $V_{\mathrm{bg}} = 0.95\,V$. Dashed lines A and B mark the constant charge state measurements performed in (b) and (c) for $B = 0\,T$. The horizontal dashed lines in (c) indicate the direct quasiparticle tunnelling at the condition $|eV_{\mathrm{sd}}| = 2\Delta$.

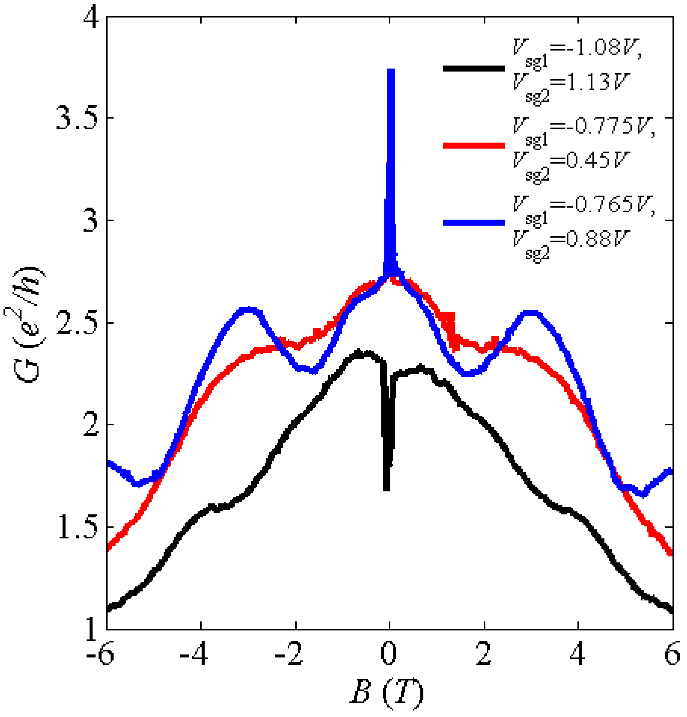

Supplemental Figure 3: Aharanov-Bohm oscillations. Magnetic evolution of the normal state $dI/dV_{\rm sd}$ at high B-fields. The sharp features at $|B| < 160\,mT$ are caused by transport with the leads in the superconducting state. The red and blue traces are measured at gate bias with both QDs ON or near resonance. The black trace is taken slightly OFF resonance resulting in smaller visibility of the AB features. In this case the conductance initially increases as Zeeman splitting shifts a Coulomb peak towards the measurement point.

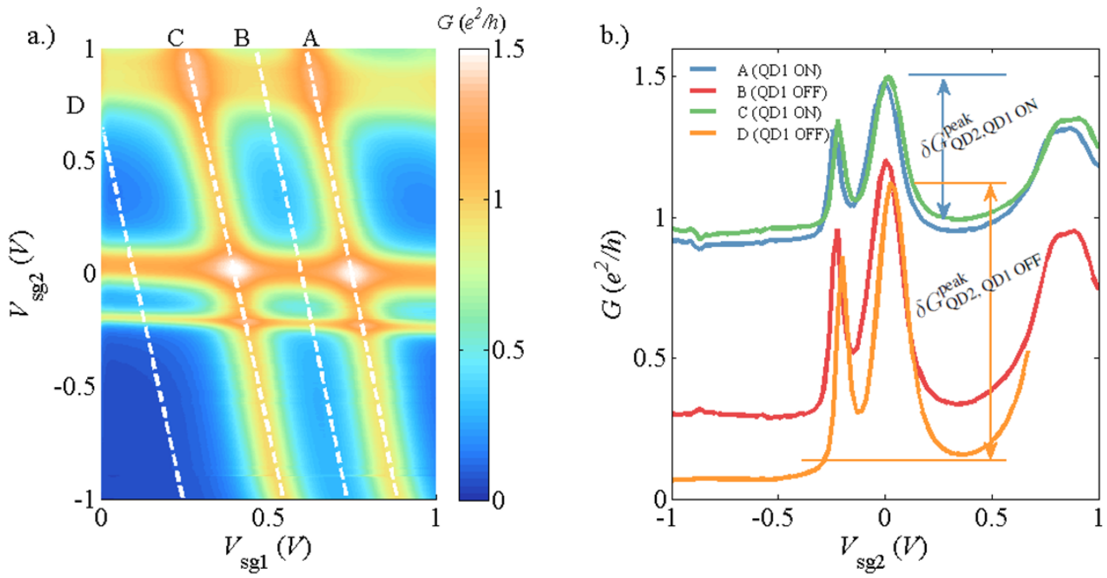

Supplemental Figure 4: Comparison of normal state conductance ON and OFF resonance with in-plane B-field. Measurements are performed with $V_{\rm bg} = 0\,V$, $V_{\rm sd} = 0\,V$ and $B = 160\,mT$ applied in-plane. (*a*) False colour plot of the differential conductance in units of e^2/h. (*b*) Extracted conductance along the lines indicated in (*a*) plotted as a function of $V_{\rm sg2}$. Lines A and C are selected such that QD1 is always ON resonance. Lines B and D are selected such that QD1 is OFF resonance.

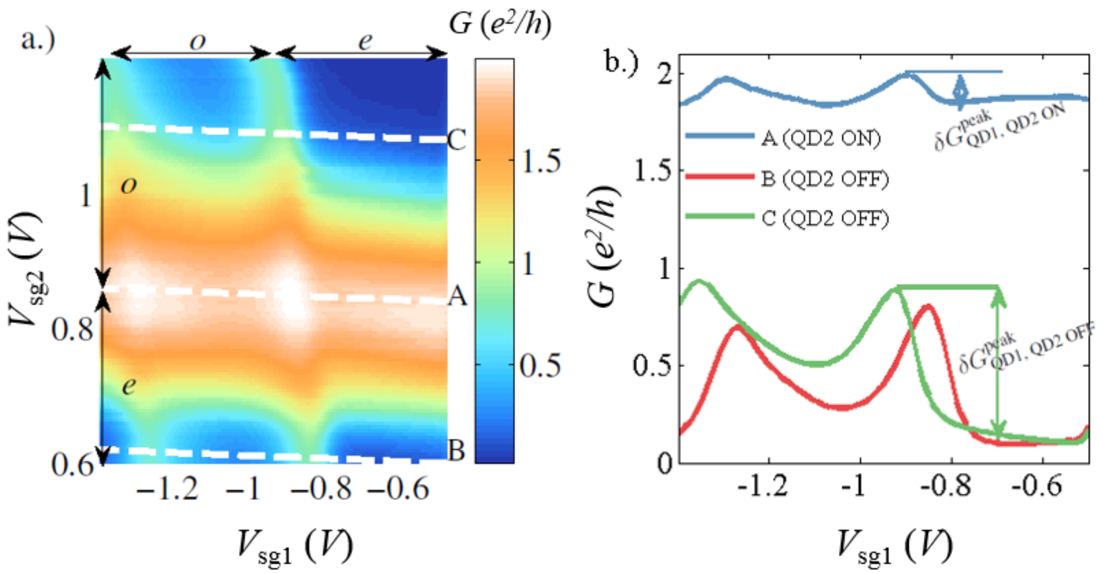

Supplemental Figure 5: Anomalous normal state transport in the region considered in Fig. 2 of main text. (*a*) Plot of the differential conductance (dI/dV_{sd}) as a function of V_{sg1} and V_{sg2} with $V_{\mathrm{sd}} = 0\,V$, $B = 160\,mT$ applied out-of-plane and $V_{\mathrm{bg}} = 0.95\,V$. (*b*) Traces extracted along lines C,D,E and F shown in (*a*). We observe that the $\delta G^{\mathrm{peak}}_{\mathrm{QD1,\,QD2\,ON}}$ approximately $70\,\%$ smaller than $\delta G^{\mathrm{peak}}_{\mathrm{QD1,\,QD2\,OFF}}$ for the most prominent features.

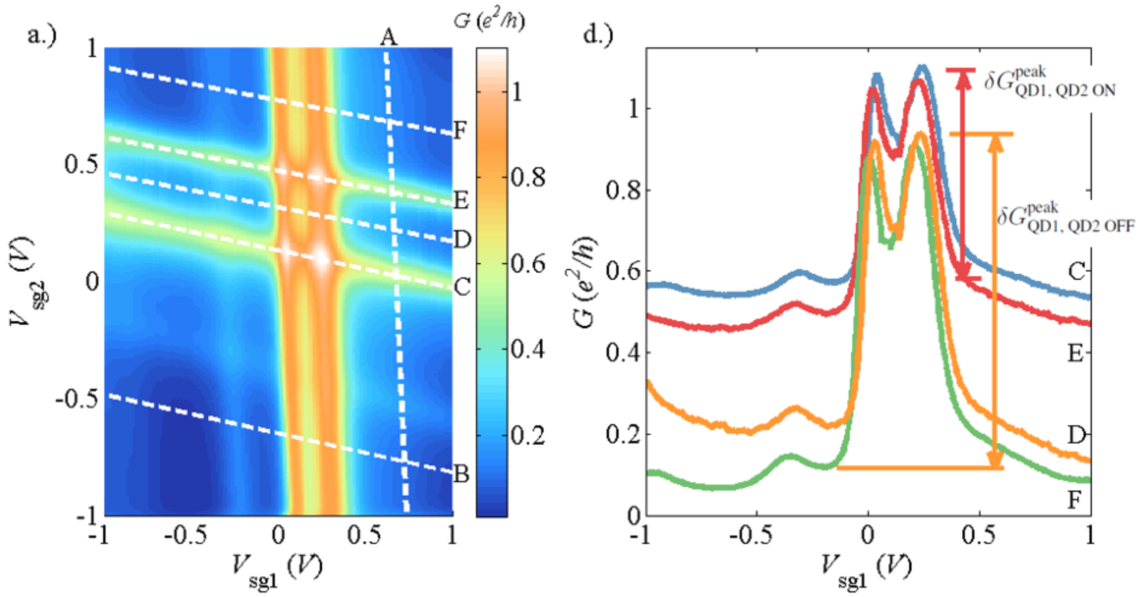

Supplemental Figure 6: Anomalous normal state transport in the region considered in Fig. 3 of main text. (a) $dI/dV_{\rm sd}$ plotted as a function of $V_{\rm sg1}$ and $V_{\rm sg2}$ with $V_{\rm bg} = 0\,V$ and $B = 160\,mT$ applied out-of-plane. Labels o and e indicate even and odd electron occupation respectively. (b) $dI/dV_{\rm sd}$ extracted along lines A, B and C in plot (a). The arrows indicate the height of the conductance peaks for QD1 when QD2 is ON and OFF resonance to highlight the anomalous conductance. We observe that the $\delta G^{\rm peak}_{\rm QD1,\,QD2\,ON}$ is approximately $30\,\%$ smaller than $\delta G^{\rm peak}_{\rm QD1,\,QD2\,OFF}$ for the most prominent features.

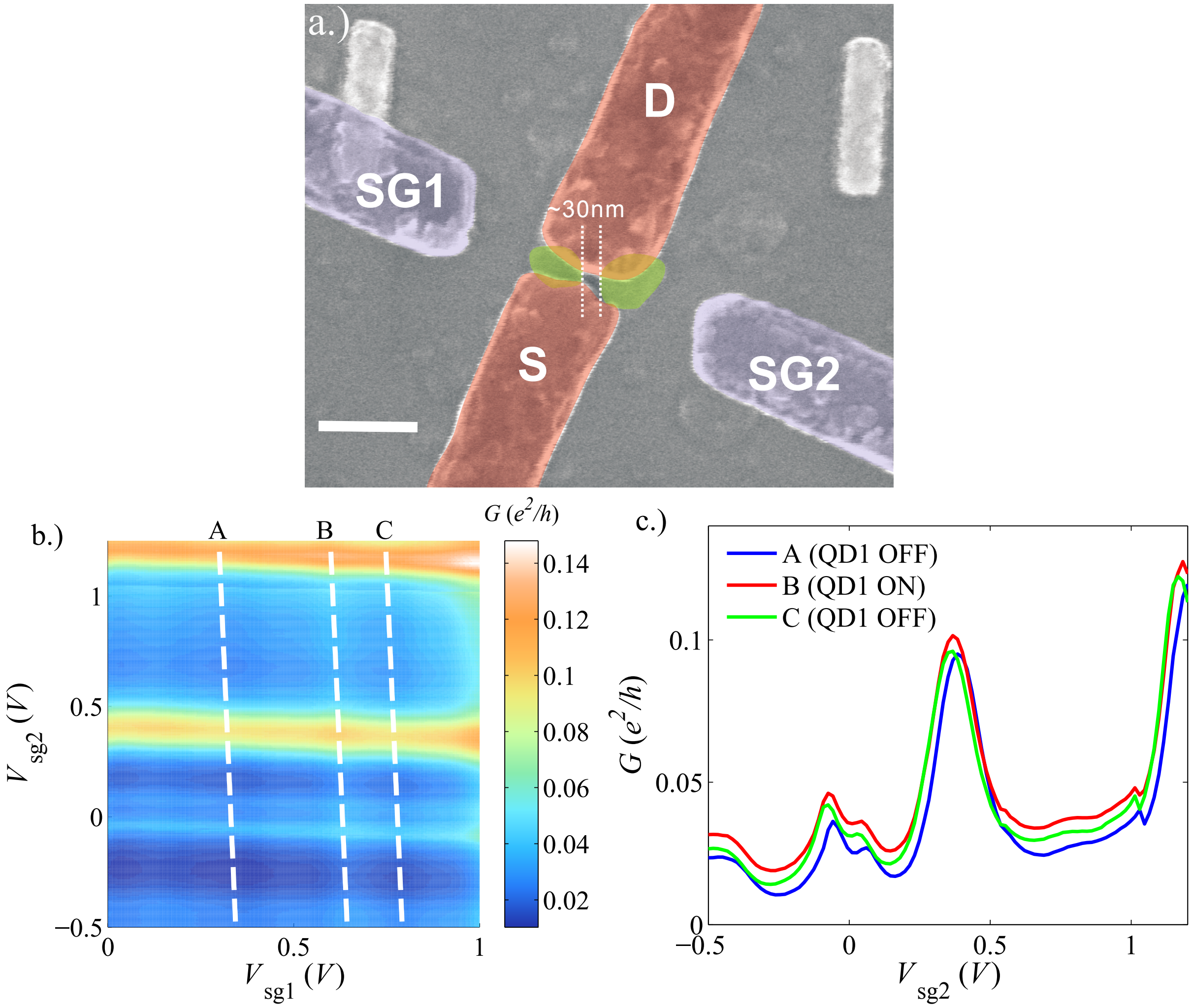

Supplemental Figure 7: Normal state transport in Device 2. (*a*) SEM image of device 2. The minimum separation between the perimeter of the two QD is $\sim 30\,nm$. The scale bar indicates $150\,nm$. (*b*) Normal state differential conductance measured as a function of $V_{\rm sg1}$ and $V_{\rm sg2}$. Measurements are performed with $V_{\rm bg} = 1\,V$ and $B = 300\,mT$ applied out-of-plane. (*c*) Normal state differential conductance traces extracted along lines A, B and C. Traces A and C are QD1 OFF resonance and Trace B is QD1 ON resonance.

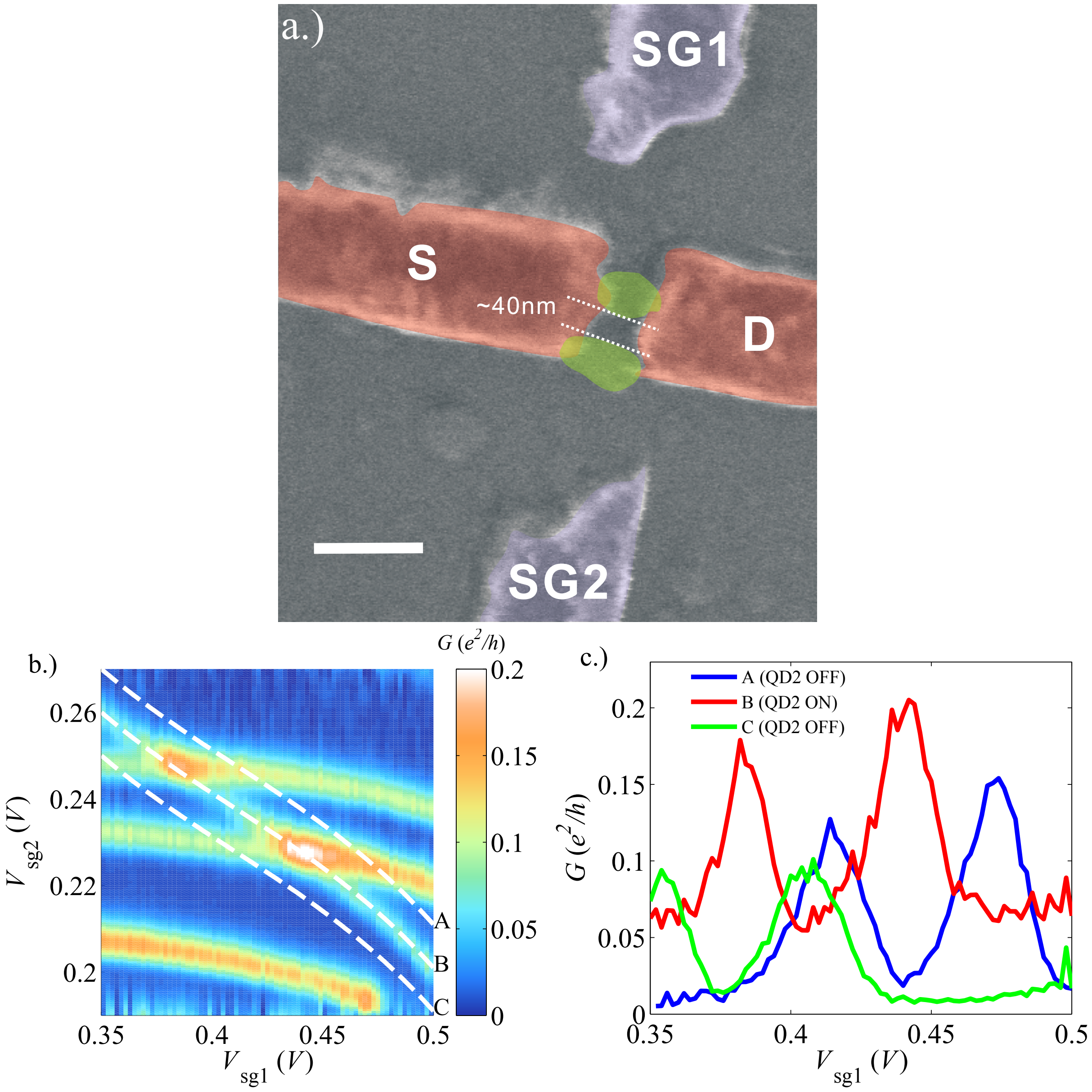

Supplemental Figure 8: Normal state transport in Device 3. (*a*) SEM image of device 3. The minimum separation between the perimeter of the two QD is $\sim 40\,nm$. The scale bar indicates $150\,nm$. (*b*) Normal state differential conductance measured as a function of V_{sg1} and V_{sg2}. Measurements are performed with $V_{\text{bg}} = 0\,V$ and $B = 300\,mT$ applied out-of-plane. (*c*) Normal state differential conductance traces extracted along lines A, B and C. Traces A and C are QD2 OFF resonance and Trace B is QD2 ON resonance.

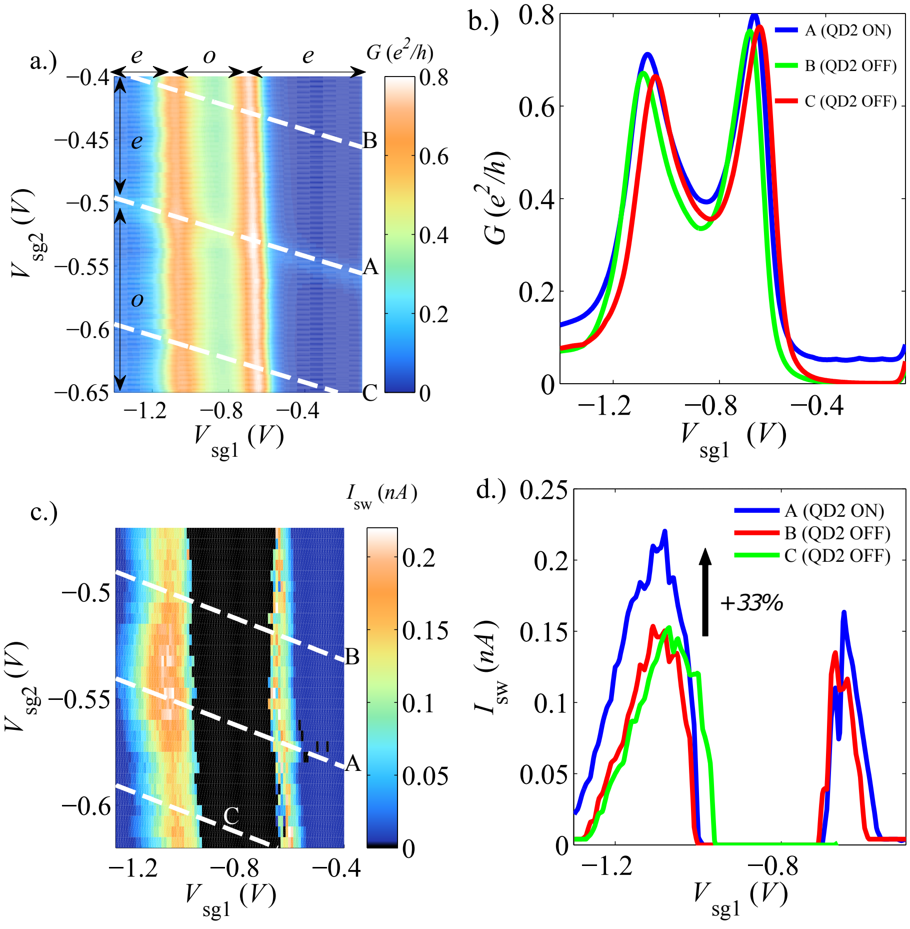

Supplemental Figure 9: Example of non-local tunnelling with weakly coupled QD2. (*a*) $dI/dV_{\rm sd}$ plotted as a function of $V_{\rm sg1}$ and $V_{\rm sg2}$ with $V_{\rm bg} = 0\,V$ and $B = 160\,mT$. Labels *e* and *o* indicate odd and even electron occupation respectively. (*b*) $dI/dV_{\rm sd}$ extracted along lines A, B and C in plot (*a*). (*c*) $I_{\rm sw}$ plotted as a function of $V_{\rm sg1}$ and $V_{\rm sg2}$ with $V_{\rm bg} = 0\,V$ and $B = 0\,T$. (*d*) $I_{\rm sw}(V_{\rm sg1})$ extracted along lines A, B and C in plot (*c*). The arrow and percentage indicates the approximate increase in $I_{\rm sw}$ when QD1 and QD2 are ON resonance.

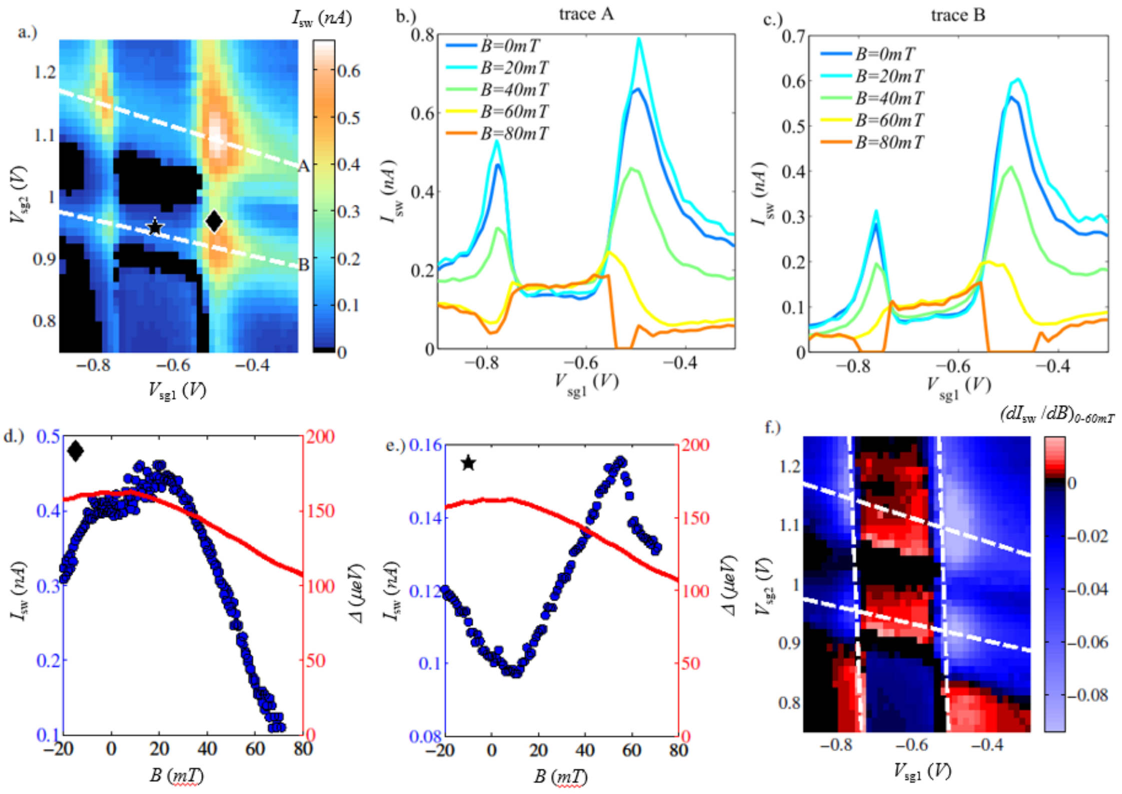

Supplemental Figure 10: Magnetic field dependence of $I_{\rm sw}$ with out-of-plane magnetic field. $I_{\rm sw}$ measured in magnetic fields applied out-of-plane. (*a*) False colour plot of the measured switching current as a function of $V_{\rm sg1}$ and $V_{\rm sg2}$ at $B = 0\,T$ and $V_{\rm bg} = 1.6\,V$. (*b*) Plots of $I_{\rm sw}$ as a function of $V_{\rm sg1}$ extrapolated along dashed line A in plot (*a*) for a range of B. (*c*) Plots of $I_{\rm sw}$ as a function of $V_{\rm sg1}$ extrapolated along dashed line d in plot (*a*) for a range of B. (*c*) Plot of evaluated $I_{\rm sw}$ as a function of B at the ♦ point indicated in plot (*a*). (*d*) Plot of evaluated $I_{\rm sw}$ as a function of magnetic field at the ⋆ point indicated in plot (*a*). (*e*) Plot of average gradient $< dI_{\rm sw}/dB >_{0-60\,mT}$ evaluated from linear fits to $I_{\rm sw}$ for magnetic fields in the range $0 - 60\,mT$. The colorscale is designed to highlight the positive and negative gradients.

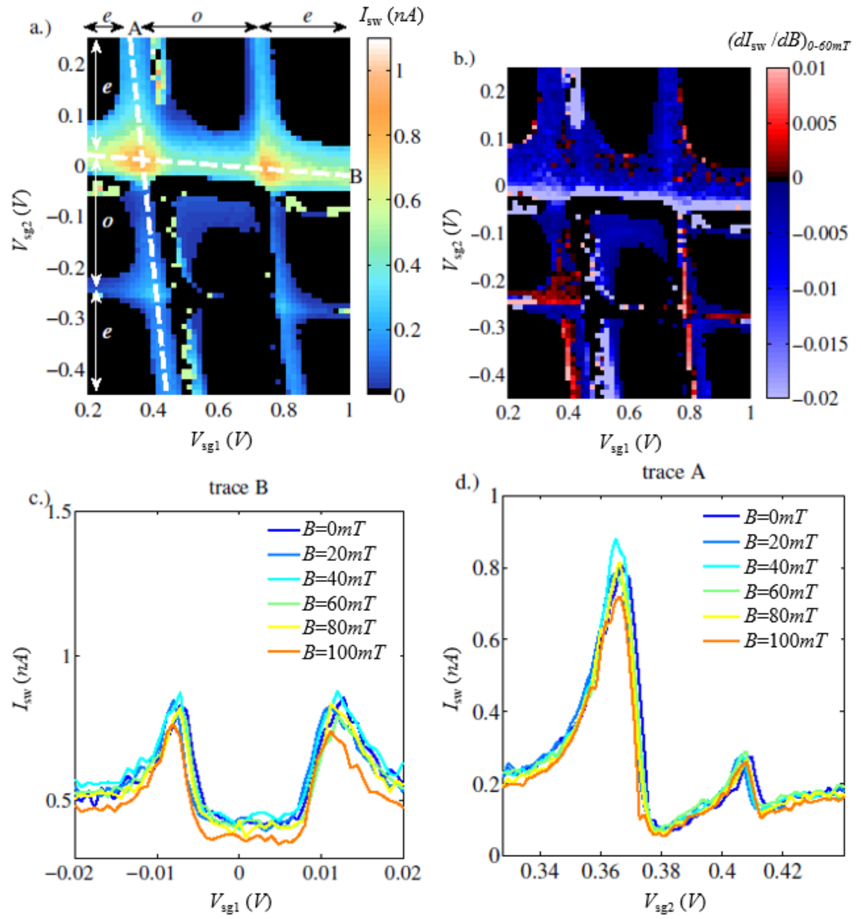

Supplemental Figure 11: Magnetic field dependence of I_{sw} with in-plane magnetic field. I_{sw} measured in magnetic fields applied in-plane. (*a*) False colour plot of the measured switching current as a function of V_{sg1} and V_{sg2} at $B = 0\,T$ and $V_{\text{bg}} = 0\,V$. (*b*) Plot of average gradient $< dI_{\text{sw}}/dB >$ evaluated from linear fits to I_{sw} for magnetic fields in the range $0 - 100\,mT$. The colorscale is designed to highlight the positive and negative gradients. (*c*) Plots of I_{sw} as a function of V_{sg1} extrapolated along dashed line C in plot (*a*) for a range of B. (*d*) Plots of I_{sw} as a function of V_{sg2} extrapolated along dashed line D in plot (*a*) for a range of B. Dashed lines in plots (*c*) and (*d*) indicate the approximate background I_{sw} measured with QD2 (QD1) ON resonance and QD1 (QD2) OFF resonance and even occupation.

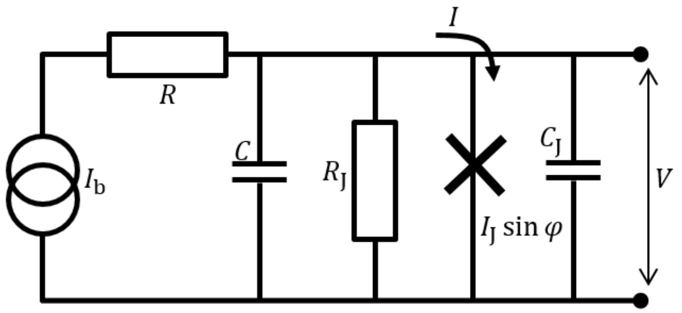

Supplemental Figure 12: Schematic of RCSJ model circuit.

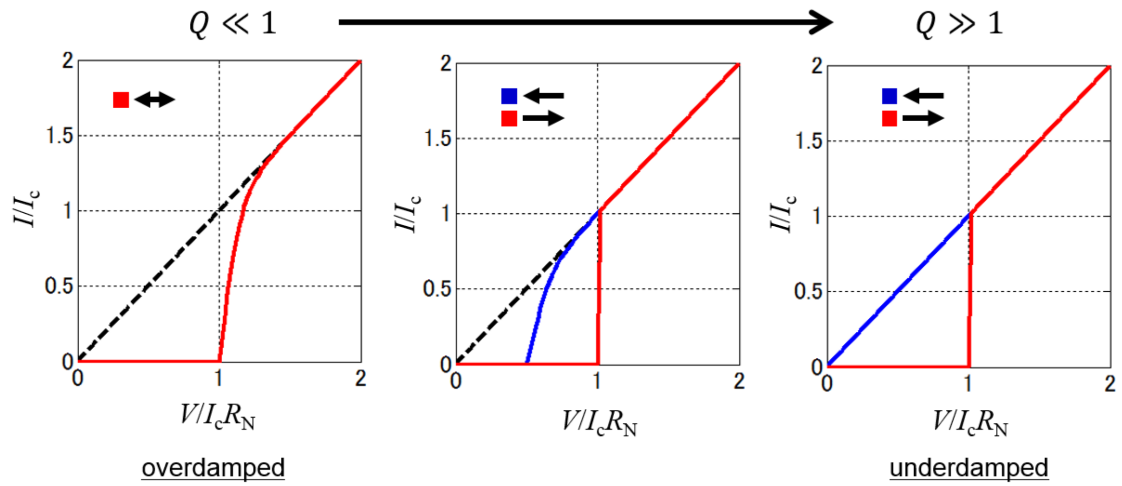

Supplemental Figure 13: $I(V)$ traces predicted by RSCJ model. Sketched example of $I(V)$ traces for different values of $\mathcal{Q}$.

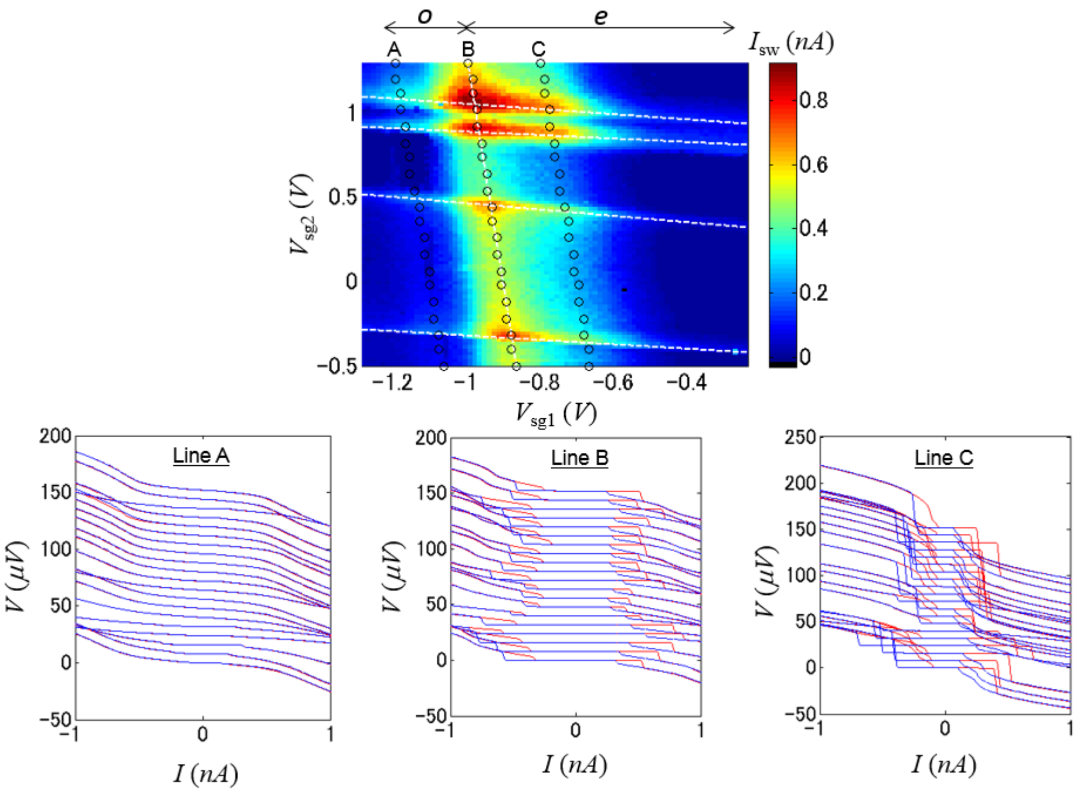

Supplemental Figure 14: Example $V(I)$ traces. Example $V(I)$ traces under different gate conditions indicated by lines A, B and C. All traces $I(V)$ are offset for clarity.

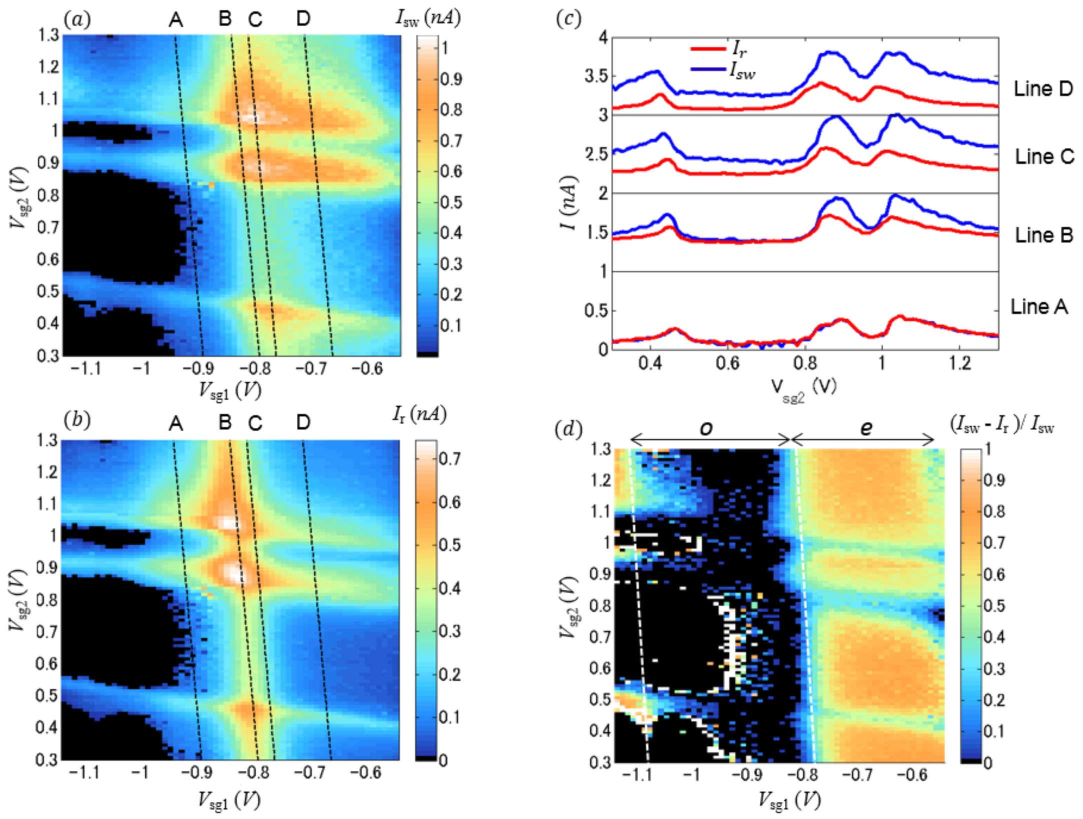

Supplemental Figure 15: Comparison of switching and retrapping currents. (a) Plot of $I_{\rm sw}$ as a function of $V_{\rm sg1}$ and $V_{\rm sg2}$. (b) Plot of $I_{\rm r}$ as a function of $V_{\rm sg1}$ and $V_{\rm sg2}$. ((c)) Plots of $I_{\rm sw}$ (blue) and $I_{\rm r}$ (red) extracted along lines A,B,C and D in (a) and (d). All traces are offset by $1\,nA$ for clarity. (d) Plot of $(I_{\rm sw} - I_{\rm r})/I_{\rm sw}$ as a function of $V_{\rm sg1}$ and $V_{\rm sg2}$. Dashed vertical white lines indicate the region in which QD1 has odd electron occupation leading to no hysteresis in the $V(I)$ trace. Labels o and e indicate odd and even electron occupation regions where known.

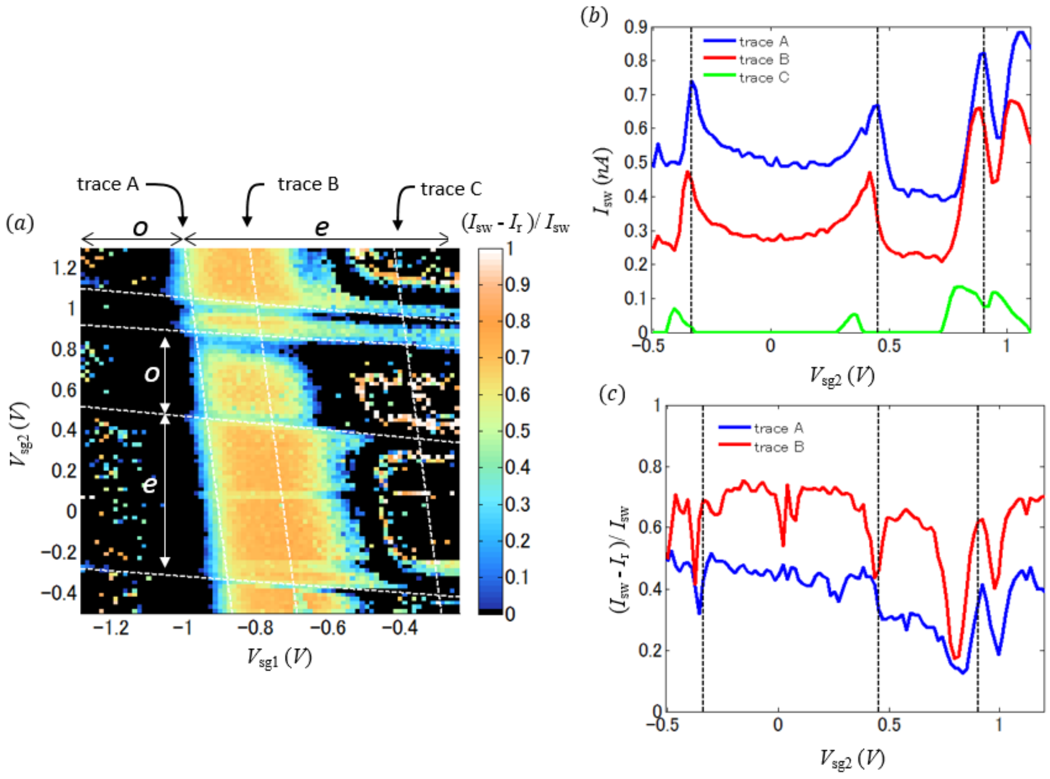

Supplemental Figure 16: Analysis of hysteresis. (*a*) Plot of $(I_{\rm sw} - I_{\rm r})/I_{\rm sw}$ as a function of $V_{\rm sg1}$ and $V_{\rm sg2}$. Labels o and e indicate odd and even electron occupation regions where known. (*b*) $I_{\rm sw}$ extracted along lines A, B and C. The vertical dashed lines indicate the position of $I_{\rm sw}$ peaks (which are coincident with the Coulomb peaks) in trace A. (*c*) $(I_{\rm sw} - I_{\rm r})/I_{\rm sw}$ extracted along lines A and B. The vertical dashed lines are as in (*b*).

Supplementary Note 1

Evaluation of an upper limit for interdot capacitance

As discussed in the main text we observe little evidence of inter-dot capacitive or tunnel coupling. While the lack of tunnel coupling maybe expected due to physical separation of the QDs some inter-dot capacitance is expected due to the close proximity. From plots of differential conductance (measured using an ac-excitation of $10\,\mu V$) as a function of V_{sg1} and V_{sg2} with the leads in the superconducting state ($B = 0\,T$) we attempt to extract estimates of the interdot capacitance from small shifts in the position of the Coulomb charging peaks for one QD when the second QD charge state is altered. Such analysis was found to be more accurate with leads in the superconducting state due to sharper transport features. An example of such data is shown in Supplementary Fig. 1 in which the position of the Coulomb peaks for QD1 have been estimated by fitting lorentzian line shapes to each peak for different constant V_{sg2}. Small deviations in the Coulomb peak positions allow us to assign an upper limit for the interdot capacitive coupling of $\sim 30\,\mu eV$ which is significantly smaller than the superconducting energy gap of the leads ($\Delta \sim 130\,\mu eV$) and therefore plays an negligible role in the transport.

Supplementary Note 2

Dissipative transport in the superconducting state

In Fig. 2 (b) and (c) we present stability plots in the superconducting state ($B = 0\,T$) measured along dashed lines A and B in Supplementary Fig. 2 (a) for which the charge state and chemical potential of QD1 and QD2 are set to be constant respectively. At low $V_{\rm sd}$ we observe clear signature of the quasi-particle tunnelling between the high density of states at the edge of the superconducting gap from which we determine $\Delta \sim 130\,\mu eV$. Additional subgap transport features indicate both multiple Andreev transport and signature of 'Yu-Shiba-Rusinov' bounds states[1,2]. The subgap transport features have been discussed for single QD junctions elsewhere[3–7] and are beyond the scope of the present study.

Supplementary Note 3

Aharanov-Bohm oscillations in the normal state

In Fig. 3 we plot the differential conductance measured at $V_{\mathrm{sd}} = 0\,V$ as a function of B applied out-of-plane for three different gate conditions. We observe periodic oscillations of the conductance which indicate the Aharanov-Bohm (AB) effect in the device with a period $\Delta B_{\mathrm{AB}} \sim 2.2 - 3.4\,T$. From the AB period we estimate the area of the AB ring ($\Delta B_{\mathrm{AB}} = \Phi_0/A$, where $\Phi_0 = h/e$ and A is the area enclosed by the AB ring) as $A \sim 1200 - 1900\,nm^2$. The fabricated nanogap is $\sim 30\,nm$ indicating that the separation of the contacts to each QD in the source/drain is $\sim 40 - 60\,nm$ which appears consistent with the proximity of the QDs indicated in SEM images (See Fig. 1 of the main text). The period of the AB oscillations varies with the charge state due to changes in the shape and extension of the wavefunction of states involved in the transport. The observation of AB oscillations at high magnetic fields indicates that coherent transport through the parallel QDs is possible despite the low coherence length in the metal contacts.

Supplementary Note 4

Additional normal state conductance data

In parallel double QDs interference effects due to inter-dot coupling have been considered in a range of theoretical and experimental works. In our system at the points where both QDs are ON resonance no anti-crossing, which is caused by the direct inter-dot coupling[8], is observed and indeed the inter-dot tunnel coupling is expected to be negligible due to the physical separation of the QDs on the substrate. We naively expect the normal state transport to mimick a simple circuit with two parallel elements, where the conductance of each element may be summed to give the conductance of the whole[9]. Contrary to this expectation we observe that the conductance is reduced from the expected value when both QDs are tuned to be ON or near resonance, as shown for a representative set of data in Supplementary Fig. 4. The anomalous conductance additivity is clear when we compare the peak conductance for QD2 with the QD1 OFF resonance $\delta G^{\text{peak}}_{\text{QD2, QD1 OFF}}$ with the peak conductance for QD2 with QD1 ON resonance $\delta G^{\text{peak}}_{\text{QD2,QD1 ON}}$. In the case in Supplementary Fig. 4 we observe that the $\delta G^{\text{peak}}_{\text{QD2,QD1 ON}}$ is approximately $50\,\%$ smaller than $\delta G^{\text{peak}}_{\text{QD2, QD1 OFF}}$. Similar results are observed in different cooldowns of the device in which the magnetic field is applied in-plane and out-of-plane (see Supplementary Figs. 5 and 6), ruling out interference caused by the AB effect.

This effect may arise from a parasitic series resistance of approximately $2-5\,k\Omega$, depending on the specific Coulomb peaks which are both ON resonance, when the transport leads are normal (note that our measurements are conducted using a 4-terminal setup eliminating the fridge lead

resistances down to the Aluminium contacts). Alternatively this effect may indicate an interaction or selectivity between the two transport paths which breaks the additivity of the conductance. In other devices with a larger separation between the perimeter of the QDs (see section which follows) we do not observe this suppressed conductance effect leading us to conclude that the proximity of the contacts to the QDs at the source and drain leads is important. The cause of the anomalous conductance is not well understood but we can speculate that interference occurs within the leads near the contacts to the QDs when separation of the QD contacts approaches the free path of carriers in the leads. We also note that similar anomalous conductance effects have been observed in Andreev entangler devices reported elsewhere[10] and it has been speculated that when one QD is ON resonance the fast charge fluctuations can have an effect to dephase the other QD and reduce conductance. Finally it is interesting to note that the supercurrent in a QD Josephson junction typically scales with the normal state resistance[11]. Therefore our observation of lower conductance in the normal state and enhanced I_{sw} in the superconducting state are at odds.

Normal state conductance has been measured for two other devices (sample 2 and sample 3) in which the separation between the contacts of the two QDs in the source and drain was larger than for the sample in the main text, Supplemenatry Figs. 7 and 8. The indicated approximate separation of the perimeters of the QDs is estimated from the SEM image. In both cases we observe the additivity of the conductance when both QDs are tuned ON resonance. In these devices we were unable to detect non-dissipative Josephson current probably due to insufficient transparency of the contacts.

Supplementary Note 5

Anomalous enhanced Josephson currents in other regions

Here we consider the case where QD1 is relatively strongly coupled to the leads resulting in a measurable local non-dissipative Josephson current while QD2 has weak coupling. In Supplementary Fig. 9 (*a*) and (*b*) we show normal state transport measurements for such a region. The transport feature for QD2 ON resonance (dashed line A) is very weak. In the superconducting state we measure a clear switching current for conditions near transport resonances with QD1. When we measure with QD2 OFF resonance (traces B and C) we associate the measured switching current with local processes through QD1. We note that I_{sw} is abruptly suppressed and becomes unmeasurable when the occupation of QD1 is tuned to odd parity. The suppression occurs on the odd-valley side of the Coulomb charging peaks for QD1 and is consistent with previous observations of a parity driven quantum phase transition in the ground state of the system between singlet and 'magnetic' double groundstates[6]. In a system with sufficient coupling in which supercurrent could be measured we would expect therefore to observe a $0-\pi$ phase transition in the phase relation of the junction in these regions. With QD1 OFF resonance we observe no Josephson current in this measurement region and therefore conclude that the local processes through QD2 are completely suppressed by the small tunnel coupling and large charging energy. When we consider the condition with both QDs ON resonance we observe an enhanced I_{sw}, shown in Supplementary Fig. 9 (*d*), indicating the presence of the non-local processes.

Supplementary Note 6

Magnetic field dependence of I_{sw}

Here we briefly discuss the observation of characteristic suppression of the measured switching current when one QDJJ is occupied by an odd electron number leading to a negative Josephson energy. Consider the data presented in Supplementary Fig. 10 (*a*) in which we focus on the crossing of a pair of Coulomb charging peaks for each QD. In Supplementary Fig. 10 (*b*) and 10 (*c*) we show traces extracted from the data in (*a*) along the dashed lines A and B. When $B = 0\,T$ we observe a clear suppression of the switching current when one QD is occupied by an odd electron number and the other QD is near resonance. This suppressed transport arises because the Josephson energy contribution for one of the QDs acquires a negative sign leading to a reduced critical current for the junction.

Further evidence of the π-junction may be observed by applying a magnetic field $(B||z)$ as shown in Supplementary Figs. 10 (*b-e*). Despite the small size of the loop we observe that for small magnetic fields in some regions the measured I_{sw} increases with increasing B-field while in other regions I_{sw} decreases. Specific example are shown for the ◆ and $\star$ points in (*a*) in Supplementary Figs. 10 (*d*) and (*e*) respectively. By taking an average derivative in the range $B = 0 - 60\,mT$, $\langle dI_{\text{sw}}/dB\rangle_{B=0-60mT}$ we observe a pattern of increasing and decreasing I_{sw} for small B-fields as shown in Supplementaru Fig. 10 (*f*). This pattern is similar to that observed for $0 - \pi$ phase transitions in QD SQUID devices such as that studied by Cleuziou *et al.*[12]. This may be understood by considering that when only one junction is occupied by an odd electron number the Josephson

energy for that junction is negative and the phase relation for that junction has a π shift relative to the other normal (or 0) junction. The total critical current can then be written as

$$I = I_1 + I_2 = I_{c1}\sin(\phi_1) + I_{c2}\sin(\phi_2 + \pi) \tag{1}$$

$$= I_{c1}\sin(\phi_1) - I_{c2}\sin(\phi_2), \tag{2}$$

where $\phi_2 = \phi_1 - 2\pi\frac{\Phi}{\Phi_0}$, $\Phi_0 = h/2e$ is the magnetic flux quantum and Φ is magnetic flux penetrating the SQUID loop. In the simplest case we consider $I_{c1} = I_{c2} = I_c$ giving

$$I = I_c\sin(\phi_1) - I_c\sin\left(\phi_1 - 2\pi\frac{\Phi}{\Phi_0}\right). \tag{3}$$

$$\tag{4}$$

When $\Phi = 0$ the current through each junction cancels and no Josephson current flows. If however $\Phi = \pi$ the critical current takes a maximum of $I_{c1} + I_{c2}$. In contrast if both junctions have the same phase relation (0 or π-junctions) the critical current takes its maximum value for $\Phi = 0$ and its minimum for $\Phi = \pi$. Due to the small size of the loop only a small decrease or increase in I_{sw} is detected before reduction due to the decrease of the superconducting gap. Note that similar measurements with $B \perp z$, shown in Supplementary Fig. 11, revealed no equivalent effects which supports the conclusion that the flux penetrating the small loop causes the magnetic field dependence.

Supplementary Note 7

Effects of the tunable dissipative environment

It is important to consider the effects of the local dissipative environment on the parallel double QD device[13–16]. The properties of the junction circuit can be captured in the resistively capacitively shunted Josephson (RCSJ) model[17]. We use the modified RCSJ model in Ref. 13 which accounts for lead capacitances and resistances by assuming the circuit shown in Supplementary Fig. 12. Here C_J and R_J are the Josephson junction capacitance and resistance respectively. R and C represent the 'on chip' extrinsic circuit resistance and capacitance respectively. The dynamics of the system are captured in the quality factor ($\mathcal{Q}$) defined as,

$$\mathcal{Q} = \frac{1}{\omega_p \left(RC + \frac{\hbar}{2e} \frac{1}{I_\mathrm{c} R_\mathrm{J}} \right)} \tag{5}$$

where ω_p is the 'plasma frequency' given as

$$\omega_p = \sqrt{\frac{2eI_\mathrm{c}}{\hbar \left(C \left(1 + \frac{\mathrm{r}}{R_\mathrm{J}} \right) + C_\mathrm{J} \right)}}. \tag{6}$$

Parameter I_c indicates the intrinsic junction critical current in the absence of fluctuations. In the limit $\mathcal{Q} \ll 1$ the dissipation is strong, the junction displays no hysteresis in the $V(I)$ traces and is said to be overdamped. If $\mathcal{Q} \gg 1$ the phase changes across the junction are fast compared with the dissipation and the junction is underdamped. In this limit the $V(I)$ traces display a hysteresis.

The retrapping current is very sensitive to the $\mathcal{Q}$ parameter as so can be used as a probe to observe the changes in the dissipation in the system. Example sketched $V(I)$ characteristics for different $\mathcal{Q}$ are shown in Supplementary Fig. 13.

In this picture we can consider that one QD in our system may act as a tunable local environment for the second QD. For example with QD A under a fixed gate condition we can tune QD B from Coulomb blockade to Coulomb peak and consider that the resistance of junction B acts as a gate variable shunt resistance which alters the phase dynamics of QD A, in effect acting as a tunable $R_{\rm J}$ in Supplementary Fig. 12. A reduced shunt resistance will result in smaller $\mathcal{Q}$ and the hysteresis in the $V(I)$ characteristic will decrease. For a single QD junction the form of the $V(I)$ can change as a function of the gate and we often observe that in regions of odd electron occupation a junction shows no hysteresis while in even occupation regions the junction shows underdamped features. Some example $V(I)$ traces under different gate conditions are shown in Supplementary Fig. 14. When measured at points along line A for which QD1 has an odd electron occupation no hysteresis is observed and the JJ appears overdamped. When QD1 has an even electron occupation (line C) we observe hysteresis and the JJ appears underdamped.

To analyse the effects of the dissipative environment in more detail we consider the ratio $(I_{\rm sw} - I_{\rm r})/I_{\rm sw}$ which gives a measure of the hysteresis in the $V(I)$ trace. Supplementary Fig. 15 shows the switching current $I_{\rm sw}$ and retrapping current $I_{\rm r}$ measured as a function of gates $V_{\rm sg1}$ and $V_{\rm sg2}$. In (*c*) we plot $I_{\rm sw}$ and $I_{\rm r}$ extracted along lines A, B, C and D in (*a*) and (*b*). In (*d*) we plot the ratio $(I_{\rm sw} - I_{\rm r})/I_{\rm sw}$. In regions with $(I_{\rm sw} - I_{\rm r})/I_{\rm sw} = 0$ the junction displays no hysteresis *or*

displays no supercurrent. In regions with $(I_{\rm sw} - I_{\rm r})/I_{\rm sw} > 0$ the junction displays an underdamped $V(I)$ characteristic. In the plot of ratio $(I_{\rm sw} - I_{\rm r})/I_{\rm sw}$ we can clearly identify the region in which QD1 has odd electron occupation despite the fact that the Coulomb peak feature at lower $V_{\rm sg1}$ is unclear in the $I_{\rm sw}$ data in (*a*) at $V_{\rm sg1} \sim -1.1\,V$.

Extracting $(I_{\rm sw} - I_{\rm r})/I_{\rm sw}$ along the resonance of QD1 (line B in Supplementary Fig. 16) we observe that there is a decrease in hysteresis close to the Coulomb peaks of QD2. The decrease is typically seen on the odd electron occupation side of the Coulomb peaks and therefore typically shifted relative to the peaks in enhanced $I_{\rm sw}$ discussed in the main text which occur on the Coulomb peak. The enhanced $I_{\rm sw}$ reported in the main text also has a different lineshape when compared with the changes in hysteresis as the enhanced $I_{\rm sw}$ with changes in $I_{\rm sw}$ spanning a wider range of gate than the regions with suppressed $(I_{\rm sw} - I_{\rm r})/I_{\rm sw}$. As the hysteresis features are not correlated with the enhanced and suppressed supercurrent we conclude that changes in the local dissipative environment caused by changes in the gate conditions cannot account for the observed features. We however cannot exclude an effect from the tunable dissipative environment as the effect if present cannot be simple extracted from the measurement data. A possible improvement for future measurements will be the engineering of a low impedance measurement environment to detect the actual critical current of the DQD JJ[18].

Supplementary References

1. Koerting, V., Andersen, B. M., Flensberg, K. & Paaske, J. Nonequilibrium transport via spin-induced subgap states in superconductor/quantum dot/normal metal cotunnel junctions. *Phys. Rev. B* **182**, 245108–245120 (2010).

2. Andersen, B. M., Flensberg, K., Koerting, V. & Paaske, J. Nonequilibrium transport through a spinful quantum dot with superconducting leads. *Phys. Rev. Lett.* **107**, 256802–256807 (2011).

3. Buizert, C., Oiwa, A., Shibata, K., Hirakawa, K. & Tarucha, S. Kondo universal scaling for a quantum dot coupled to superconducting leads. *Phys. Rev. Lett.* **99**, 136806–136809 (2007).

4. Eichler, A. *et al.* Even-odd effect in andreev transport through a carbon nanotube quantum dot. *Phys. Rev. Lett.* **99**, 126602–126605 (2007).

5. Sand-Jespersen, T. *et al.* Kondo-enhanced andreev tunneling in inas nanowire quantum dots. *Phys. Rev. Lett.* **99**, 126603–126606 (2007).

6. Kanai, Y. *et al.* Electrical control of kondo effect and superconducting transport in a side-gated InAs quantum dot josephson junction. *Phys. Rev. B* **82**, 054512–054519 (2010).

7. Lee, E. J. H. *et al.* Zero-bias anomaly in a nanowire quantum dot coupled to superconductors. *Phys. Rev. Lett.* **109**, 186802–186806 (2012).

8. Hatano, T. *et al.* Manipulation of exchange coupling energy in a few-electron double quantum dot. *Phys. Rev. B* **77**, 241301–241304(R) (2008).

9. Qin, H. *et al.* Probing coherent electronic states in double quantum dots. *phys. stat. sol. (c)* **1**, 2094–2110 (2004).

10. Das, A. *et al.* High-efficiency cooper pair splitting demonstrated by two-particle conductance resonance and positive noise cross-correlation. *Nature Communications* **3**, 1165 (2012).

11. Kanai, Y. *et al.* Control of supercurrent in a self-assembled inas quantum dot josephson junction by electrical tuning of level overlaps. *App. Phys. Lett.* **100**, 202109–202111 (2012).

12. Cleuziou, J., Wersdorfer, W., Bouchiat, V., Ondarçuhu, T. & Monthioux, M. Carbon nanotube superconducting quantum interference device. *Nature Nanotechnology* **1**, 53–59 (2006).

13. Jarillo-Herrero, P., van Dam, J. & Kouwenhoven, L. Quantum supercurrent transistors in carbon nanotubes. *Nature* **439**, 953–956 (2006).

14. Liu, G., Zhang, Y. & Lau, C. N. Gate-tunable dissipation and "superconductor-insulator" transition in carbon nanotube josephson junctions. *Phys. Rev. Lett.* **102**, 016803–016806 (2009).

15. Jørgensen, H., Novotný, T., Grove-Rasmussen, K., Flensberg, K. & Lindelof, P. Critical current 0-π transition in designed josephson quantum dot junctions. *Nanoletters* **7**, 2441–2445 (2007).

16. Eichler, A. *et al.* Tuning the josephson current in carbon nanotubes with the kondo effect. *Phys. Rev. B* **79**, 161407–161410(R) (2009).

17. Tinkham, M. *Introduction to Superconductivity* (McGraw-Hill, 1996).

18. Chauvin, M. *et al.* Crossover from josephson to multiple andreev reflection currents in atomic contacts. *Phys. Rev. Lett.* **99**, 067008–067011 (2007).
